# Symbolic Play and Novel Noun Learning in Deaf and Hearing Children: Longitudinal Effects of Access to Sound on Early Precursors of Language

**DOI:** 10.1371/journal.pone.0155964

**Published:** 2016-05-26

**Authors:** Alexandra L. Quittner, Ivette Cejas, Nae-Yuh Wang, John K. Niparko, David H. Barker

**Affiliations:** 1 Department of Psychology, University of Miami, Coral Gables, FL, United States of America; 2 Department of Otolaryngology, University of Miami Miller School of Medicine, Barton G. Kids Hear Now Cochlear Implant Family Resource Center, Miami, FL, United States of America; 3 School of Medicine, Johns Hopkins University, Baltimore, MD, United States of America; 4 Department of Otolaryngology-Head and Neck Surgery, University of Southern California, Los Angeles, CA, United States of America; 5 Department of Child and Adolescent Psychiatry, Brown University, Providence, RI, United States of America; University of Salamanca- Institute for Neuroscience of Castille and Leon and Medical School, SPAIN

## Abstract

In the largest, longitudinal study of young, deaf children before and three years after cochlear implantation, we compared symbolic play and novel noun learning to age-matched hearing peers. Participants were 180 children from six cochlear implant centers and 96 hearing children. Symbolic play was measured during five minutes of videotaped, structured solitary play. Play was coded as "symbolic" if the child used substitution (e.g., a wooden block as a bed). Novel noun learning was measured in 10 trials using a novel object and a distractor. Cochlear implant vs. normal hearing children were delayed in their use of symbolic play, however, those implanted before vs. after age two performed significantly better. Children with cochlear implants were also delayed in novel noun learning (median delay 1.54 years), with minimal evidence of catch-up growth. Quality of parent-child interactions was positively related to performance on the novel noun learning, but not symbolic play task. Early implantation was beneficial for both achievement of symbolic play and novel noun learning. Further, maternal sensitivity and linguistic stimulation by parents positively affected noun learning skills, although children with cochlear implants still lagged in comparison to hearing peers.

## Introduction

Developmental scientists have successfully applied dynamic systems theory to our understanding of early language acquisition [1,2], proposing that the interactions between a child’s internal processes and the environment foster the emergence of early language skills [3,4]. During periods of malleability, perturbations of the system facilitate reorganization of these processes, via systemic phase shifts that can increase functional adaptation in a specific context. Early childhood deafness, prior to the critical period for language acquisition, is one example of a major perturbation of the dynamic systems involved in early language development [2], and results in significant language delays [5,6,7,8]. Restoration of auditory input via cochlear implantation provides an opportunity to examine how the language system responds to changes in auditory information. Additionally, this auditory deprivation severely limits young deaf children’s access to ambient sources of linguistic information (i.e., adults, television), making the child more dependent on their parents for language stimulation. Our major research questions concern how two environmental influences, timing of auditory restoration and quality of parent-child interactions, affect the development of early language learning in young deaf children prior to and following cochlear implantation.

Decades of research have demonstrated that children born with severe-to-profound hearing losses do not develop oral language skills at a rate similar to their hearing peers [5]. Restoration of auditory information via cochlear implantation may serve to reorganize this system to *facilitate and extend* the period for acquisition of early precursors of language [9,10]. A cochlear implant stimulates the auditory nerve, bypassing the defective cochlea, to provide auditory information to the developing brain [11]. It has been shown to increase auditory input and thus, improve the development of oral language [7]. Children implanted earlier (i.e., before age 2) show better language development than those implanted later [6]. A few studies have examined the influence of cochlear implantation and its timing on young children's acquisition of linguistic skills, such as symbolic play and novel noun learning [9, 12, 13, 14], however, none have examined these skills from pre to post-implantation or over time. Our aim was to examine how enhanced auditory input would affect the development of these two early language skills in a large, national cohort of deaf children before and three years after implantation.

In addition, our previous research has shown that maternal sensitivity, coupled with parental linguistic stimulation, significantly influenced the developmental trajectory of oral language several years post-implantation [15]. Hearing parents of deaf children who exhibited higher levels of sensitivity, warmth, and positive regard had children who performed substantially better on standardized language measures four years after implantation. Further, the magnitude of this effect was equal to the benefits of early versus later implantation on language development [6], and this effect was strongest for children of parents who showed high maternal sensitivity and linguistic stimulation [15]. This is the first study to examine the influence of parental sensitivity on the development of symbolic and novel noun learning.

### Symbolic Play in Deaf Children and Hearing Children

In typically developing children, the use of language to label objects emerges during the same period as the use of symbols in play (e.g., a peg to represent a person) [16]. There are several different types of "pretend" or "symbolic" play; however, in our study we used a strict definition of symbolic play as a substitution of an object in a child's solitary play (e.g., a block as a bed). Unfortunately, little is known about these processes in young children with hearing loss.

Gregory and Mogford studied six deaf and six hearing children 15 to 30 months of age and found that children with delayed language showed appropriate representational play with realistic toys, but were less likely than hearing children to engage in more abstract, symbolic play [17]. In a study of similar-aged children [18], deaf children were delayed relative to hearing peers in their pretend play during structured tasks with a caregiver. Importantly, delays in language development led to slower development of pretend play. However, because pretend play was measured in the context of parental mediation (e.g., parents played with their child), it did not provide a test of the child’s spontaneous, *solitary* use of symbols during play. In contrast, two studies found no differences in the frequency of symbolic play in two-year old deaf and hearing dyads [19,20].

In sum, studies comparing the emergence of symbolic play in deaf and hearing children are contradictory, with half documenting delays and half reporting no delays. Prior studies have been plagued by methodological limitations, such as small samples, cross-sectional designs, mediation of play by parents or experimenter, and different definitions of symbolic play. Notably, these studies were conducted prior to the widespread use of neonatal hearing screening and cochlear implants. Our study overcame these limitations by utilizing the largest, nationally representative sample of young deaf children, studied prior to and for three years following cochlear implantation.

## Novel Noun Learning in Deaf and Hearing Children

By the end of the second year of life, typically developing children rapidly learn new vocabulary, and by three years of age, they are highly skilled word learners. Many studies have focused on the content and size of children’s lexicons and have shown strong relationships between the rate of noun acquisition and the development of receptive and expressive language. Research has also examined the processes that underlie children’s word acquisition [1,10], showing that by 18 months of age young hearing children are able to reliably identify and map the name of novel objects based on its shape, and can generalize this to other similarly shaped objects [21]. Novel noun learning is also associated with the development of oral language and may be more susceptible to disruptions in linguistic input than symbolic play.

Given deaf children's limited access to linguistic input, they are likely to acquire novel noun learning more slowly. Studies of vocabulary development in deaf children have consistently found delays related to size and growth, and learning new vocabulary is dependent on previous vocabulary knowledge [22,23,24].

Several studies have examined novel noun learning in hearing-impaired children. Recent studies of young deaf children using cochlear implants (CIs) [13,14] generally indicated that these children did not identify novels nouns as rapidly as hearing controls. Houston and colleagues tested 25 deaf children with implants, ages 22–40 months, all with at least one-year implant experience, in comparison to 23 normal hearing children. When compared to hearing controls, children implanted by 14 months of age, and those with residual hearing, performed better than older, late-implanted children. They also found a significant relationship between performance on this word learning task and measures of language. In a study of oral preschoolers using CI’s (n = 24) and normal hearing children (n = 47), word learning was worse in the CI vs. hearing group when age-matched. However, when matched on vocabulary (Peabody Picture Vocabulary Test), their performance did not differ. They did not find an association between word learning and age at implantation or maternal education. Unfortunately, they did not test the association between word learning and language.

In studies of older children with hearing loss, Gilbertson and Kamhi compared hearing-impaired children, ages 8 to 11 with mild to moderate losses, to a hearing group of 5 to 10 year olds [25]. In the novel noun learning trials, no significant differences were found on the single syllable word, however, hearing-impaired children performed consistently worse on nouns with two or more syllables and required more learning trials than their peers. More recently, Stelamachowicz and colleagues assessed novel noun learning in children six to nine years of age, asking them to identify the novel word from four panels [9]. Hearing-impaired children scored significantly lower than hearing children (41% vs. 60% correct). Similar results have been found in younger, hearing-impaired children ages three to seven years [23,26]. Slightly more than half of the children succeeded in mapping the novel nouns.

In a large study of CI and hearing children, ages 6 to 12 years, tests of novel word learning, receptive vocabulary and speech perception were administered [12]. Novel word learning scores were significantly below those of the hearing controls, however, children implanted at younger ages and those with better hearing thresholds performed better. All of these studies have concluded that novel noun learning emerges more slowly for children with hearing loss and is related to their receptive vocabulary. No study has examined novel noun learning before and after implantation or using a longitudinal design.

In sum, lack of auditory input limits the linguistic environment for young deaf children, leading to deficits in symbolic play, novel noun learning and oral language. Restoring auditory information via a cochlear implant allows us to examine how early language skills change as a function of the developmental timing of the implant. Parental behaviors may also strongly influence the development of symbolic play and novel noun learning in this population. Thus, our primary aim is to evaluate the development of both symbolic play and novel noun learning in deaf children pre to post implantation in comparison to hearing peers. Additional aims included: 1) to compare the effects of chronological age and age at implantation on the acquisition of these skills, and 2) to evaluate the effects of maternal sensitivity and linguistic stimulation on their development. To test our aims, we used data from the Child Development after Cochlear Implantation (CDaCI), the largest most nationally representative study of the effects of cochlear implants on children’s development.

## Materials and Methods

Participants were recruited from six clinical implant centers and two preschools that enrolled normal hearing children [27]. Children ages 5 months to 5 years were enrolled in the study prior to receiving their implant and were then followed for three years after implantation. Inclusion criteria for children in the CDaCI study were: 1) age under 5 years, 2) severe-to-profound sensorineural hearing loss (implant candidates only), and 3) parents committed to educating the child in spoken English. Exclusion criteria included significant cognitive impairment (i.e., a Bayley Mental or Motor score of less than 70 or Leiter International Performance Scale–Revised (Leiter-R) score of less than 66). Children with minor cognitive deficits were included to increase the generalizability of the findings to a broader population of children receiving cochlear implants. Normal hearing (NH) children were selected to be similar to the cochlear implant (CI) children in terms of age and gender. All participants were assessed at Baseline (prior to implantation for the CI group) and every six months (from point of activation for CI group) for three years, and annually thereafter. Institutional review boards at all centers and preschools approved the study protocol (i.e., University of Miami, Johns Hopkins University, University of Michigan, University of North Carolina-Chapel Hill, University of Texas at Dallas, & House Ear Institute). Written consent was obtained from all parents or legal guardians to collect data from both caregivers and the children who participated. Data for these analyses were drawn from assessments at Baseline, 6, 12, 24, and 36 months post-implantation.

### Participants

The CDaCI cohort consisted of 188 deaf and 97 hearing children. Demographic information is presented in Table 1. Deaf children had a mean age of 2.2 years, SD = 1.2, and hearing children had a mean age of 2.3, SD = 1.1 at Baseline. Among the deaf children, age of onset of hearing loss varied between birth and 3.6 years, M = 0.2 years, SD = 0.6, and the mean Pure Tone Average (PTA4) for the better ear was 105.84, SD = 17.4. Hearing loss was diagnosed at a mean age of 0.9 years, SD = 0.9, but for a majority of these children (56%), the onset of hearing loss was at birth. Average length of hearing aid use prior to implantation was 1.1 years, SD = 1.0. We examined whether children had a unilateral or bilateral implant. At point of implantation, 4 out of 188 were bilateral recipients; at 12 months post-implant, 14 children had bilateral implants; at 24 months, 20 children had bilateral implants, and at 36 months, 35 children had bilateral implants. In terms of the type of CI, most children (55%) had a Cochlear device, 32% had an Advanced Bionics device, and 13% had a MEDEL device.

**Table 1:** Demographics of the CDaCI Cohort.

Characteristic	CI (n = 188)	NH (n = 97)
Age in years (SD)	2.2 (1.2)	2.3 (1.1)
Age of onset of hearing loss (years)	0.2 (0.6)	–
Pure-tone average (PTA4; better ear)	105.84 (17.4)	–
Age at diagnosis (years)	0.9 (0.9)	–
Age at first hearing aid use (years)	1.1 (0.9)	–
Length of hearing aid use (years)	1.1 (1.0)	–
*Onset of hearing loss % (n)*		
Sudden	6% (11)	–
Progressive	34% (64)	–
Congenital	56% (105)	–
*Cause of hearing loss % (n)*		
Genetic	28% (53)	–
Other	15% (27)	–
Unknown	57% (108)	–
*Gender % (n)*		
Male	48% (90)	38% (37)
Female	52% (98)	62% (60)
*Race % (n)*		
White	75% (140)	79% (77)
African-American	9% (17)	11% (11)
Asian	6% (11)	2% (2)
Other/No response	10% (20)	7% (7)
*Ethnicity % (n)*		
Hispanic	20% (37)	9% (9)
Non-Hispanic	77% (145)	89% (86)
*Communication mode* ^a^ *% (n)*		
Speech	20% (37)	–
Sign	19% (35)	–
Simultaneous/Speech Emphasis	19% (35)	–
Simultaneous/Sign Emphasis	6% (12)	–
Other / Undecided	37% (69)	–
Parents’ education** % (n)		
< High school	7% (14)	5% (5)
High school grad	14% (26)	2% (2)
College	78% (147)	92% (89)
Parents’ income** % (n)		
< $15,000	8% (15)	5% (5)
$15–29,999	12% (22)	4% (4)
$30–49,999	22% (42)	6% (6)
$50–74,999	16% (31)	14% (14)
$75–100,000	14% (26)	13% (13)
$100,000 +	16% (31)	51% (49)
*Full-term pregnancy % (n)*		
Yes	86% (161)	78% (76)
No	11% (21)	11% (11)

* p < .05.

** p < .01.

a children in this study were cochlear implant candidates and all parents committed to teach their children spoken English. These categories, therefore, represent the primary communication mode used prior to enrollment in this study.

Comparisons of the demographic data between the CI and NH cohorts indicated they were similar on child age, gender, and racial composition. However, the CI sample had a higher proportion of Hispanic children, lower parental education, and lower income, likely due to the geographical locations of the sites from which CI vs. NH children were recruited (see Table 1).

### Procedures

After an initial medical screening for children in the CI group, a Baseline assessment was performed by a speech and language pathologist two to four weeks prior to cochlear implant surgery. The assessment was conducted during two half-day appointments to lessen fatigue for the child and family. During the first day, parents completed demographic and self-report measures of communication and behavior, and children were assessed with language measures, cognitive tests, and an audiological exam. On the second day, children participated in videotaped structured and unstructured tasks with and without parents, and parents completed psychosocial measures about their children. All measures, including those assessing language, were conducted in spoken English. Parents in the CI group received a $100 honorarium annually, travel stipends if required, and extended warranties for the implants as reimbursement for their time and effort; parents in the hearing group received the same honorarium. All written and videotaped materials were de-identified to ensure confidentiality. CI candidates were scheduled for surgery 2–4 weeks after the Baseline assessment, with a return visit 4–6 weeks later for implant activation.

### Measures

#### Symbolic Play

Symbolic play was examined using procedures developed by Bornstein and colleagues [19]. Children engaged with one of four play sets during 5 minutes of videotaped, structured solitary play. During this task, parents were asked to sit near their child, but refrain from interacting with them. To facilitate this, parents were given questionnaires to complete during the task and were asked to redirect their child back to the toys, without labeling any objects. Toys were selected to encourage a variety of different play behaviors, ranging from simple exploration to complex symbolic play. Toy Set I was used during the Baseline assessment; Toy Sets II—IV were randomized throughout the study visits (see Table 2 for a description of toy sets). Data were collected for this task at Baseline, 6, 12 and 24 months.

**Table 2:** Description of Symbolic Play Toy Sets.

Toy Set	Materials
Set I (Baseline Assessment)	Doll, pillow, wooden block, blanket
Set II	Cylinder, airplane, chair, house
Set III	Doll, dish, spoon, cup, bag of pom poms, poker chips, and foam shapes
Set IV	Toy bridge in three pieces, triangle block, rectangle block

Symbolic play was coded using an adaptation of Belsky and Most’s coding scheme [28] to categorize children’s play behaviors using Observer Video-Pro Version 5.0. Play was coded continuously as a dichotomous variable (i.e., symbolic vs. non-symbolic), with start and end times recorded. For example, in Toy Set I, using the wooden block as a bed for a doll was coded as symbolic play. Interrater reliability for presence of symbolic play was calculated using percent agreement. Two independent coders rated 20% of the tapes, with percent agreement ranging from 80 to 89%, indicating good reliability. Previous studies have indicated that childhood deafness affects sustained attention [29], which may influence the amount of time children engaged in play with these toys. Differences in the amount of time spent on the task, which are unrelated to symbolic representation, could potentially distort the results based on the time spent in different types of play. Thus, we used the strict definition of symbolic play as "substitution" (i.e., at least one instance of the child using an abstract object as a realistic object; a block as a bed). Using this strict definition, we expected that children who evidenced some oral language would be able to complete this task.

#### Noun Classification

To examine the rate at which children with and without hearing learned novel nouns, we utilized a task developed by Smith and colleagues [1]. Children were eligible to complete this task once they had a vocabulary of 50 or more words as measured by parent report on the MacArthur Communicative Development Inventory [30]. This task consisted of 10 trials. Data were collected on this task at Baseline, 6, 12, 24, and 36 months.

**Fig 1:**
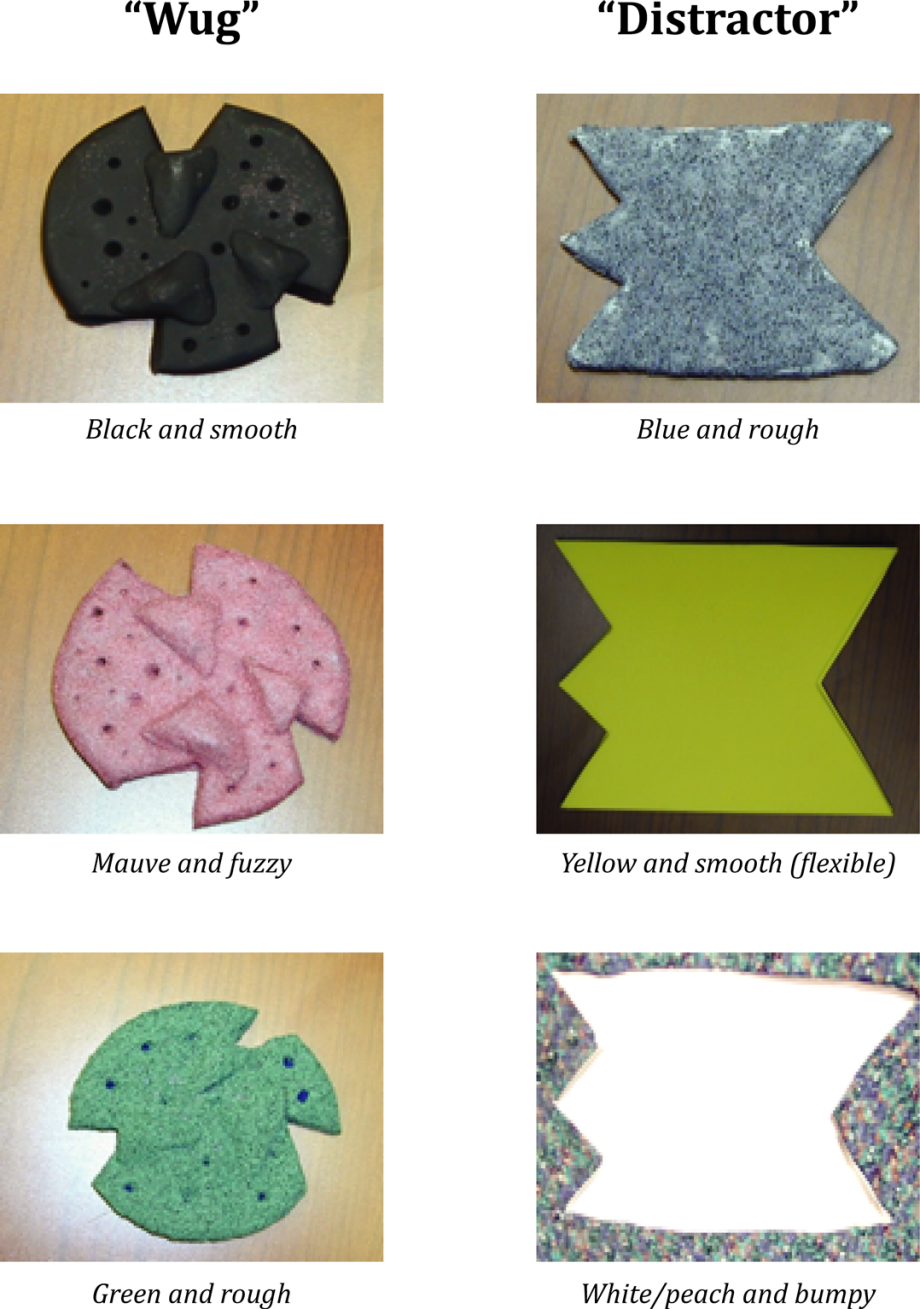
Noun class stimuli. Illustrates the exemplar "wug" and the distracter that were used during the 10 trials. Remaining stimuli were used during the generalization trials and varied by shape, color, and texture as illustrated above.

After a brief warm-up (naming simple objects, such as “cup” and “ball”) a novel object (called a “wug”) was presented to the child along with a distracter object. The shapes of these 2 objects were quite different (Fig 1). As part of the training, the experimenter named and presented the exemplar "wug" and the distracter to the child, separately, on three occasions. After the third presentation of the objects, the child was asked to select the exemplar three times ("Where is the wug?"), alternating its physical location with the distractor as part of the 10 trials. Next, a series of generalization trials followed in which the wug retained its shape, but was presented in a different color and texture (e.g., from black to green and/or smooth to fuzzy), with alternating locations. The child’s ability to learn the novel noun was quantified as 0 or 1 depending on the correct identification of the wug. Achievement of novel noun learning was considered 8 or more correct answers across the 10 trials. Success in this task was coded as a dichotomous variable (i.e., 0 or 1).

#### Quality of Parent-Child Interactions

Quality of parent-child interactions were measured using the NICHD Early Childcare Study tasks [31,32]. Parent-child interactions were videotaped during several structured and unstructured activities. Maternal Sensitivity (MS) and Linguistic Stimulation (LS) were coded during three standardized, well-established tasks lasting 20 minutes: Free Play, Problem-Solving, and Art Gallery. During the unstructured Free Play task, the parent and child were directed to “play as you would at home.” In the Problem-Solving task, the parent and child spent five minutes completing two puzzles, one that was easy for the child and one that was difficult. During the Art Gallery task, the parent and child looked at a series of five art posters mounted on the walls of the playroom at different heights and discussed the posters for five minutes [33].

Interactions were coded using the NICHD study codes, including the MS composite, which consists of four subscales: Sensitivity/Responsivity, Respect for Child’s Autonomy, Positive Regard, and Hostility. Trained observers coded the interactions using a 7-point rating scale (1 = very low; 7 = very high). The Sensitivity/Responsivity scale reflects the degree to which the mother expresses positive regard and emotional support to the child. Respect for Child’s Autonomy assesses whether the mother recognizes and respects the child’s individuality, motives, and perspectives during the session. Positive Regard rates the amount of positive feelings directed to the child (e.g., parent watches attentively, praises child). Hostility is reversed-coded and reflects the parent’s expression of anger toward or rejection of the child.

LS was coded using a 7-point scale developed by a research team of psychologists and speech/language pathologists at the University of Miami [15]. This scale measures the amount and quality of stimulation that facilitates functional auditory and linguistic skill development. For example, parents scoring high on this scale would use a variety of techniques to move the child along the listening hierarchy, such as exposing the child to sound, asking for behavioral or verbal responses, and linking sounds and objects in the environment. All coders completed an extensive training process, including weekly group meetings in which difficult tapes were coded and reviewed. Independent coding of tapes followed feedback from the authors, along with coding of several previously coded tapes to reach a criterion of 80% reliability. Periodic checks of coders’ scores were performed every 3 months to ensure consistency and good reliability. One-fifth of all tapes (n = 1425) were randomly selected for each assessment point and coded (i.e., full 20 minutes of video). Reliability of the MS and LS scales ranged from 0.78 to 0.84. Two trained coders independently rated 20% of the videotapes, yielding good interrater reliability (intraclass correlation coefficient: for MS, 0.79–0.93 [mean, 0.86]; for LS, 0.73–0.89 [mean, 0.80]).

#### Language Measures

MacArthur-Bates Communicative Development Inventories (CDI) [30]. The CDI is a widely used parent report measure of children’s language. Section I of the Words and Sentences Inventory, which measures vocabulary for children ages 16 to 30 months, was used. This inventory has also been used with preschool children who have language impairment [34]. Words were only recorded if they were understood or spoken orally. The words inventory has good internal consistency (.96).

Reynell Developmental Language Scales (RDLS) [35]. The RDLS are commonly used, well-validated language scales for children ages one to seven years. They have been used with deaf and hearing children [36,37]. This test also provides explicit instructions regarding adaptation of test administration for deaf children. The measure consists of a Verbal Comprehension and Expressive Language scale. Both scales have acceptable split-half reliability coefficients across age groups ranging from .74 to .93. Children’s scores can be compared to normative data to produce either standard scores or language age.

Estimate of Language Age. Language age was estimated for each participant using the same approach used by Quittner et al. [15]. First only scores that were within the usable range of the two CDI and two RDLS subscales were translated into age-equivalent scores. Scores that were at the documented floor or ceiling of each subscale were eliminated from the dataset. Language age for each assessment was then estimated by averaging the age-equivalent scores from available subscales. This approach helped to extend the floor of the RDLS by adding information from the CDI.

### Analytic strategy

Time-to-event analyses with interval censoring [38] were used to model the age at which children in the CI and NH cohorts achieved criterion on the two primary outcomes: Symbolic Play (Symbolic Play Criterion: presence of symbolic representation during play) and Noun Classification (Noun Classification Criterion: 8 correct responses on the 10 classification trials). Interval censoring was used because we did not observe the exact age at which children achieved the criterion on each task. In place of exact age, the analyses used intervals defined by the children’s ages at the assessment when they were first observed to meet criterion and their ages at the previous assessment. For children who met criterion at the Baseline assessment, the lower bound of the interval was placed at age 6 months (additional lower bounds were tested including birth and 3 months with no substantive changes in results).

A Non-parametric maximum likelihood estimator was used to estimate the survival function for each outcome. The estimation was conducted using the EM-ICM algorithm developed by Wellner and Zahn [39] and implemented using the R “Icens” package [40]. Survival functions were stratified by hearing status, age at implantation, and quality of parent-child interactions (3 videotapes were uncodeable because of equipment malfunction and were dropped from the analyses of parent-child interactions). Hazard ratios (HR) were estimated using proportional hazards regression models for interval censored data [41] and implemented using the R “intcox” package [42] with bootstrapped standard errors and confidence intervals. These models controlled for Baseline characteristics available for both NH and CI cohorts, including maternal education, child age at Baseline, child IQ and gender (models using only the CI cohort and adjusting for CI-specific covariates are presented in S1 and S2 Tables). Adjusted survival curves and median survival times were calculated from the regression models. Delay between the CI and NH cohorts was calculated as the difference between median survival times.

The relationships among age at implantation, quality of parent-child interactions, and oral language have been previously examined using language age as an outcome [15]. We sought to replicate these findings using the interval-censored time-to-event models, thus providing a benchmark to compare children’s performance on the symbolic play and novel noun learning tasks. For the replication, we used achieving an oral language age of 24 months as our benchmark.

Two approaches were used to examine the associations among language age, symbolic play, and novel noun learning. First the associations between these measures were assessed at Baseline using Spearman rank-order correlations, where symbolic play and novel noun learning were defined as the proportion of participants achieving criterion at Baseline. The second approach examined the longitudinal relationships among the measures by calculating Pearson correlation coefficients among the ages at which participants achieved each of the three criteria. All analyses were performed using R version 3.1.2.

## Results

### Oral Language Age

It was expected that children with oral language would be able to complete the symbolic play task. For the CI cohort, 164 (92%) children had a language age less than 24 months at Baseline, despite their chronological age of 2.07 (1.38 to 3.15) years and 15 (8%) had a language age greater than two years, with a mean age of 4.06 (3.72 to 4.40) years. For the NH cohort, 40 (42%) were below two years and 55 (58%) were greater than two years. By three years post-implantation (5th assessment), 76% of the CI and 100% of the NH participants met the Oral Language Criterion—a language age of at least 24 months.

**Fig 2:**
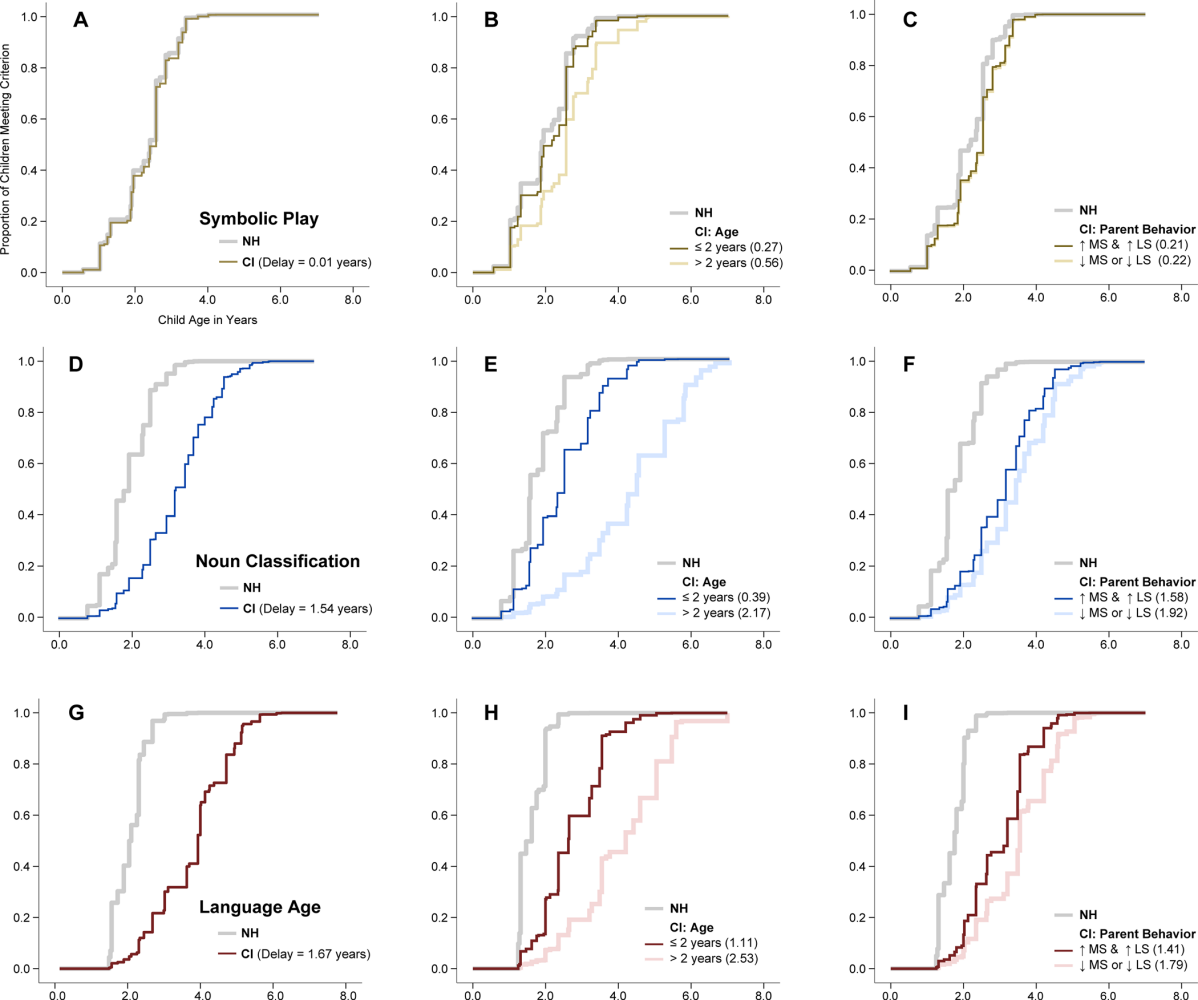
Predicted proportions of CI and Hearing children meeting criterion by age and parenting behavior. Compares acquisition of symbolic play and noun classification by hearing status, age at implantation, and maternal sensitivity and linguistic stimulation. The top panel illustrates symbolic play, the middle panel illustrates noun classification, and the bottom panel illustrates language age. Estimated Survival Functions. A) Symbolic Play by hearing status; B) Symbolic Play by implantation age; C) Symbolic Play by Parent Behavior; D) Noun Classification by hearing status; E) Noun Classification by implantation age; F) Noun Classification by parent behavior; G) Language Age by hearing status; H) Language Age by implantation age; I) Language Age by parent behavior. Delays represent the difference in adjusted median survival times with the NH cohort as the reference group.

The time-to-event models for oral language (Tables 3–5; Fig 2) showed results similar to those reported in our prior study [15]. Namely, the CI vs NH cohort showed marked delays in oral language, children implanted younger were more likely to meet criterion than children implanted after age 2, and children of parents who demonstrated high maternal sensitivity and high linguistic stimulation were more likely to meet criterion than those whose parents demonstrated either low maternal sensitivity or low linguistic stimulation.

**Table 3:** Cox Regressions comparing CI and hearing groups.

	Oral Language		Symbolic Play		Noun Class	
	Estimate (SE)	HR	Estimate (SE)	HR	Estimate (SE)	HR
**Group Comparison**						
Hearing Status *(0 = Hearing; 1 = CI)*	-2.65 (0.24)*	0.07 (0.04 to 0.11)	-0.07 (0.21)	0.93 (0.61 to 1.42)	-1.77 (0.29)*	0.17 (0.10 to 0.30)
**Control Variables**						
Maternal Education (years)	0.34 (0.05)*	1.41 (1.27 to 1.56)	0.11 (0.05)*	1.11 (1.01 to 1.22)	0.21 (0.06)*	1.23 (1.10 to 1.37)
Child’s Age at Enrollment (years)	-0.79 (0.10)*	0.45 (0.55 to 0.38)	-0.63 (0.12)*	0.53 (0.42 to 0.67)	-0.80 (0.09)*	0.45 (0.38 to 0.53)
Child’s Gender *(0 = Male; 1 = Female)*	0.12 (0.15)	1.16 (0.84 to 1.50)	0.71 (0.17)*	1.84 (1.31 to 2.59)	0.46 (0.17)*	1.59 (1.12 to 2.24)
Child’s IQ *(in standard deviation units)*	0.13 (0.07)	1.14 (1.00 to 1.29)	-0.05 (0.06)	0.95 (0.85 to 1.08)	0.17 (0.06)*	1.18 (1.05 to 1.32)

SE = Standard Error; HR = Hazard Ratio.

* *p* < .05.

**Table 4:** Cox Regressions comparing children implanted prior to vs. after age two in relation to hearing peers.

	Oral Language		Symbolic Play		Noun Class	
	Estimate (SE)	HR	Estimate (SE)	HR	Estimate (SE)	HR
**Contrasts**						
Hearing all ages	2.15 (0.28)*	8.58 (4.85 to 15.17)	-0.17 (0.21)	0.84 (0.56 to 1.26)	0.93 (0.22)*	2.54 (1.65 to 3.91)
Implanted before age 2	Reference group	–	Reference group	–	Reference group	–
Implanted after age 2	-1.46 (0.20)*	0.23 (0.16 to 0.35)	-0.75 (0.20)*	0.47 (0.32 to 0.70)	-1.71 (0.22)*	0.18 (0.11 to 0.27)
**Control Variables**						
Maternal Education *(years)*	0.27 (0.04)*	1.31 (1.20 to 1.43)	0.11 (0.05)	1.01 (0.92 to 1.10)	0.17 (0.05)*	1.19 (1.08 to 1.31)
Child’s Gender (*0 = Male; 1 = Female)*	-0.06 (0.15)	1.06 (0.80 to 1.40)	0.39 (0.17)*	1.47 (1.07 to 2.04)	0.38 (0.17)*	1.46 (1.05 to 2.01)
Child’s IQ (*in standard deviation units*)	0.13 (0.06)*	1.14 (1.01 to 1.28)	-0.04 (0.06)	0.97 (0.86 to 1.09)	0.17 (0.06)*	1.18 (1.05 to 1.34)

SE = Standard Error; HR = Hazard Ratio.

* *p* < .05.

**Table 5:** Cox Regressions comparing Maternal Sensitivity and Linguistic Stimulation.

	Oral Language		Symbolic Play		Noun Class	
	Estimate (SE)	HR	Estimate (SE)	HR	Estimate (SE)	HR
**Contrasts**						
Hearing controls	2.43 (0.26)*	11.33 (6.75 to 19.01)	0.40 (0.22)	1.48 (0.97 to 2.28)	1.74 (0.29)*	5.67 (3.23 to 9.97)
High Maternal Sensitivity and High Linguistic Stimulation	Reference Category	–	Reference Category	–	Reference Category	–
Either Low Maternal Sensitivity or Low Linguistic Stimulation	-0.64 (0.21)*	0.53 (0.35 to 0.80)	0.02 (0.22)	1.02 (0.67 to 1.56)	-0.37 (0.20)	0.69 (0.47 to 1.03)
**Control Variables**						
Maternal Education *(years)*	0.25 (0.05)*	1.28 (1.16 to 1.41)	0.09 (0.05)	1.09 (0.99 to 1.05)	0.15 (0.06)*	1.16 (1.03 to 1.31)
Child’s Age at Enrollment *(years)*	-0.76 (0.08)*	0.47 (0.40 to 0.55)	-0.65 (0.12)*	0.52 (0.42 to 0.66)	-0.80 (0.09)*	0.45 (0.38 to 0.53)
Child’s Gender (*0 = Male; 1 = Female*	0.03 (0.14)	1.03 (0.78 to 1.36)	0.58 (0.17)*	1.79 (1.27 to 2.51)	0.43 (0.19)*	1.54 (1.06 to 2.26)
Child’s IQ (*in standard deviation units*)	0.16 (0.06)*	1.16 (1.04 to 1.33)	-0.06 (0.06)	0.94 (0.84 to 1.06)	0.17 (0.06)*	1.19 (1.06 to 1.34)

SE = Standard Error; HR = Hazard Ratio.

* *p* < .05.

### Symbolic Play

#### Symbolic Play and Oral Language

Across both CI and NH cohorts, the correlation between Baseline language age and Baseline symbolic play was *r* = .42. For children who did not meet the symbolic play criterion at Baseline, correlation between the age when they achieved the task and the age when they achieved oral language was *r* = .34.

#### Symbolic Play and Hearing Status

Fewer CI (37%, *n* = 69) than NH (49%, *n* = 47) children met the criterion for symbolic play at Baseline; similarly, fewer CI (77%, *n* = 144) than NH (94%, *n* = 91) children had successfully met criterion by the end of the fourth assessment point. Proportional hazards models indicated non-significant differences between the CI and NH groups (Table 3; Fig 2). The model also suggested that maternal education, child age at enrollment, and child gender were related to task completion. Mothers with more education, children who were younger at enrollment, and females were more likely to meet criterion.

#### Symbolic Play and Age at Implantation

The proportional hazard model indicated children implanted prior to age two were more likely to meet criterion than those implanted after age two (Table 4; Fig 2), with non-significant differences between children implanted prior to age two and the NH cohort. The model also suggested that females were more likely to meet criterion.

#### Symbolic Play and Quality of Parent-Child Interactions

Next, we evaluated the effects of the quality of parent-child interactions on the achievement of symbolic play. Based on our prior study [15], we hypothesized that parents exhibiting both high MS and LS would have children who were more likely to achieve symbolic play. We compared children whose parents were above the median on both MS and LS to those below the median on one *or* both of these measures (Table 5; Fig 2). Results indicated non-significant differences in the timing of symbolic play based on parent-child interactions.

### Noun Classification

#### Noun Classification and Oral Language

Across both CI and NH cohorts, the correlation between Baseline language age and Baseline novel noun learning was *r* = .57. For children who did not meet the novel noun criterion at Baseline, correlations between the age when they achieved the task and the age when they achieved oral language was *r* = .83.

#### Noun Classification and Hearing Status

As predicted, fewer CI (1%, *n* = 2) than NH (44%, *n* = 43) children met the 80% criterion at Baseline; similarly, fewer CI (70%, *n* = 119) than NH (96%, *n* = 93) children had successfully met the criterion by the end of the fifth assessment point. Time-to-event analyses indicated that children in the CI group were less likely to accomplish the task than children in the NH group (Table 3; Fig 2). The model also suggested that maternal education, age at enrollment, child gender, and IQ were related to task completion. Mothers with more education, children who were younger at enrollment, females, and children with a higher IQ score at Baseline were more likely to complete the task.

#### Noun Classification and Age at Implantation

Time-to-event analyses indicated children implanted prior to age two were significantly more likely to accomplish the novel noun learning task than those implanted after age two (Table 4; Fig 2). We also expected that deaf children implanted before age two would develop noun class skills at rates similar to their hearing peers. Contrary to our expectations, children implanted before age two still showed delays in this task when compared to hearing peers.

#### Noun Classification and Quality of Parent-Child Interactions

Similar to symbolic play, we evaluated the effects of the quality of parent-child interactions on noun classification by comparing children of parents who were above the median in both MS and LS to the remainder of the CI and hearing cohorts (Table 5; Fig 2). Results indicated that children whose parents were high on both were somewhat more likely to achieve criterion than children whose parents were low on both, HR = 1.45 (0.97 to 2.13). Despite the small positive effects of the quality of parent-child interactions, children in the CI cohort continued to be delayed in achieving noun classification when compared to their hearing peers.

## Discussion

Overall, our results indicated that deaf children with cochlear implants were delayed compared to hearing children in their achievement of symbolic play and novel noun learning. These delays were larger and more persistent for novel noun learning than symbolic play, suggesting that learning novel nouns may be more influenced by the linguistic environment than engaging in symbolic play. For symbolic play, maternal education and child age at enrollment were significantly related in both cohorts to task completion. Results were similar for noun learning, with the addition of child IQ as a significant predictor. We did not find a delay in symbolic play among children implanted before the age of two (median delay = 0.27 years), but did find a delay in children implanted after two (median delay = 0.56 years). The small effect that is only apparent in older children may help explain the inconsistent results in previous studies, as some studies may not have been powered to detect a small effect [19,20]. Our results for novel noun learning are consistent with prior studies in deaf children, which found that this skill emerges more slowly and is related to language development [12]. In contrast to symbolic play, the delay in novel noun learning was considerable (median delay = 1.54 years), suggesting that this skill may be more reliant on early linguistic information than symbolic play.

Similar to findings for oral language [6,15,43,44,7], the timing of implantation appears to have a substantial impact on the development of early language skills. Deaf children who were implanted earlier (before age two) and thus, had auditory input during the sensitive period for language development, developed symbolic play at a rate similar to normal hearing peers. These results are consistent with studies of brain plasticity and reorganization which have indicated that sensory input must be established early in life, optimally before age two, to adequately process sensory information leading to better developmental outcomes, such as speech perception and oral language [45,46,47,48].

Novel noun learning also developed more rapidly for children implanted earlier vs. later but in contrast to symbolic play, it did not develop at rates similar to hearing peers (median delay = 0.39 years). This result is consistent with the findings for oral language using the CDaCI cohort suggesting that children in the CI cohort do not appear to "catch up" to their hearing peers. The reasons for this delay are not known but could be related to deficits in other cognitive and behavioral domains that are related to language development, such as sustained attention, visual selective attention, and externalizing behavior problems [49,29,50,51].

We also evaluated how the quality of parent-child interactions influenced the development of these skills. Maternal sensitivity and linguistic stimulation had no effects, either individually or combined, on the emergence of symbolic play. The limited influence of parental input, coupled with the modest “boost” in the growth of these representational skills provided by earlier implantation, indicates that these skills are more "hard wired" in development and not as strongly influenced by the linguistic environment. In contrast, on the more complex task of noun classification, maternal sensitivity and linguistic stimulation were significantly associated with improved performance. Despite this positive effect, children with CIs continued to be behind their hearing peers in acquisition of novel noun learning, which is likely related to their ongoing delays in oral language [5,6]. Together these findings suggested that both symbolic play and novel noun learning are influenced by the linguistic environment, but it appears that environmental effects (i.e., timing of implantation, quality of parent-child interactions) have stronger influences on novel noun learning than symbolic representation.

This study had a few limitations. First, the actual ages at which children met criterion on the outcomes were not observed. For a significant number of participants, the actual criterion age occurred before their baseline assessment and for the remainder, the study design only provided 6 to12-month assessment windows in which to observe the actual age at which they met criterion. While the analytic strategy reduced bias for comparisons between the CI and NH cohorts, the estimated median delays are imprecise and caution should be used when comparing these delays to other samples. Another limitation was the unequal matching of parents’ education in the CI and NH groups. Parents of children with CIs had lower levels of education than parents in the typically developing group. However, parental education was controlled in our analyses. Analyses focused on comparisons between both NH and CI cohorts, and thus, did not adjust for baseline characteristics specific to the CI-cohort. Supplementary analyses on the CI cohort (S1 and S2 Tables) showed that adjusting for these factors did not substantially alter the conclusions of this study.

Finally, this study relied on a natural experiment (early childhood deafness) that was theorized to primarily affect language development in children with normal cognition. This was supported by the IQ results, which yielded average scores for the deaf sample [6]. However, there could be other subtle neurological effects of deafness that were not measured or had not yet emerged in this young cohort [52].

Given our results, which indicated that earlier implantation is beneficial for the development of both language and symbolic play, the advent of universal newborn screening in the US should facilitate identification of children with severe-to-profound sensorineural hearing losses. Since 90% of deaf children are born to hearing parents, parents are strongly motivated to use oral language and communication with their children, which is best facilitated by early implantation [53]. Further, earlier cochlear implantation is likely to confer multiple benefits not only in terms of language, but also in strengthening parent-child relationships and interactions, reducing parental stress, and improving attention and behavior [54,6,29,55]. Thus, early intervention for young children will likely prove beneficial for multiple domains of functioning.

Future directions for this research would included measuring how the duration and intensity of auditory-verbal therapy affects early language outcomes. In addition, given the robust findings that parental sensitivity is related to both novel noun learning and other language outcomes [6,15], interventions that combine parental sensitivity with auditory-verbal therapy should be developed and tested.

## Supporting Information

S1 File Analytic Dataset and Data Dictionary. Data for oral language, symbolic play, novel noun learning, and maternal sensitivity. File includes all hearing and demographic variables.
(XLSX)

S1 Table CI-Specific Model for Age of Implantation. Model using only the CI cohort and adjusting for CI-specific covariates for oral language, symbolic play, and noun class.
(DOCX)

S2 Table CI-Specific Model for Maternal Sensitivity. Model for oral language, symbolic play, and noun class, controlling for CI-specific covariates.
(DOCX)
