# Supplementary material for: Symbolic Play and Novel Noun Learning in Deaf and Hearing Children: Longitudinal Effects of Access to Sound on Early Precursors of Language
Source: PLoS One. 2016 May 26;11(5):e0155964. doi: 10.1371/journal.pone.0155964 (PMC4882020; doi:10.1371/journal.pone.0155964)
Supplement: S1 Table — Model using only the CI cohort and adjusting for CI-specific covariates for oral language, symbolic play, and noun class. (DOCX) [file pone.0155964.s002.docx]

| **Hazard Ratio (95% CI)** | **Oral Language** | | **Symbolic Play** | **Noun Class** |
| --- | --- | --- | --- | --- |
| Implanted before age 2 | Reference group | | Reference group | Reference group |
| Implanted after age 2 | 0.21 (0.11 to 0.38)* | | 0.40 (0.23 to 0.69)* | 0.19 (0.06 to 0.61)* |
| Maternal Education (years) | | 0.21 (0.11 to 0.38) | 1.01 (0.90 to 1.13) | 1.01 (0.90 to 1.14) |
| Female Gender | | 1.04 (0.67 to 1.62) | 1.02 (0.66 to 1.58) | 1.04 (0.67 to 1.62) |
| Child’s IQ | | 1.00 (0.86 to 1.16) | 1.00 (0.85 to 1.16) | 1.00 (0.85 to 1.17) |
| Pure-tone average (better ear) | | 0.99 (0.97 to 1.00)* | 1.02 (1.01 to 1.03)* | 0.99 (0.98 to 1.00)* |
| Length of hearing aid use (years) | | 1.04 (0.45 to 2.41) | 1.08 (0.47 to 2.46) | 1.02 (0.44 to 2.39) |
| *Onset of hearing loss* |  | |  |  |
| Sudden | Reference group | | Reference group | Reference group |
| Progressive | 1.85 (0.45 to 7.54) | | 2.35 (0.26 to 21.12) | 2.25 (0.57 to 8.94) |
| Congenital | 2.06 (0.52 to 8.17) | | 3.03 (0.34 to 27.11) | 1.80 (0.45 to 7.24) |
| *Cause of hearing loss* |  | |  |  |
| Genetic | Reference group | | Reference group | Reference group |
| Other | 1.36 (0.55 to 3.32) | | 0.96 (0.43 to 2.16) | 0.55 (0.24 to 1.25) |
| Unknown | 1.16 (0.64 to 2.12) | | 0.74 (0.46 to 1.21) | 0.84 (0.48 to 1.49) |
| Bilateral Implantation | 2.06 (0.95 to 4.48) | | 1.26 (0.62 to 2.56) | 1.07 (0.46 to 2.48) |
| *Device Type* |  | |  |  |
| Advanced Bionics | Reference group | | Reference group | Reference group |
| Cochlear | 0.94 (0.54 to 1.63) | | 0.86 (0.5 to 1.46) | 0.98 (0.58 to 1.67) |
| MEDEL | 0.63 (0.28 to 1.39) | | 0.52 (0.23 to 1.16) | 0.62 (0.25 to 1.54) |

**S1 Table. CI-Specific Model for Age of Implantation.**

* *p* < .05.
