# Supplementary material for: Symbolic Play and Novel Noun Learning in Deaf and Hearing Children: Longitudinal Effects of Access to Sound on Early Precursors of Language
Source: PLoS One. 2016 May 26;11(5):e0155964. doi: 10.1371/journal.pone.0155964 (PMC4882020; doi:10.1371/journal.pone.0155964)
Supplement: S2 Table — Model for oral language, symbolic play, and noun class, controlling for CI-specific covariates. (DOCX) [file pone.0155964.s003.docx]

| **Hazard Ratio (95% CI)** | **Oral Language** | **Symbolic Play** | **Noun Class** |
| --- | --- | --- | --- |
| High Maternal Sensitivity and High Linguistic Stimulation | Reference group | Reference group | Reference group |
| Either Low Maternal Sensitivity or Low Linguistic Stimulation | 0.44 (0.25 to 0.78)* | 0.91 (0.53 to 1.55) | 0.67 (0.36 to 1.25) |
| Maternal Education (years) | 1.02 (0.90 to 1.15) | 1.01 (0.90 to 1.13) | 1.00 (0.89 to 1.14) |
| Child’s Age at Enrollment (years) | 1.01 (0.84 to 1.20) | 1.01 (0.84 to 1.22) | 1.00 (0.84 to 1.20) |
| Female Gender | 1.07 (0.68 to 1.70) | 1.03 (0.65 to 1.63) | 1.02 (0.66 to 1.58) |
| Child’s IQ | 0.99 (0.85 to 1.16) | 1.00 (0.85 to 1.17) | 1.00 (0.84 to 1.19) |
| Pure-tone average (better ear) | 1.00 (0.99 to 1.02) | 1.03 (1.01 to 1.05)* | 1.00 (0.99 to 1.01) |
| Age at diagnosis (years) | 1.03 (0.97 to 1.09) | 0.98 (0.89 to 1.08) | 1.04 (0.98 to 1.11) |
| Age at first hearing aid use (years) | 0.93 (0.87 to 1.00)* | 1.00 (0.91 to 1.11) | 0.93 (0.87 to 1.00)* |
| Length of hearing aid use (years) | 1.07 (0.44 to 2.61) | 1.04 (0.43 to 2.53) | 1.04 (0.41 to 2.64) |
| *Onset of hearing loss* |  |  |  |
| Sudden | Reference group | Reference group | Reference group |
| Progressive | 1.82 (0.23 to 14.3) | 1.11 (0.2 to 6.17) | 1.7 0(0.31 to 9.39) |
| Congenital | 1.66 (0.21 to 13.2) | 1.46 (0.23 to 9.12) | 1.37 (0.23 to 8.33) |
| *Cause of hearing loss* |  |  |  |
| *Genetic* | Reference group | Reference group | Reference group |
| *Other* | 1.27 (0.53 to 3.03) | 0.83 (0.35 to 1.97) | 0.49 (0.18 to 1.3) |
| *Unknown* | 1.17 (0.66 to 2.08) | 0.68 (0.41 to 1.13) | 0.82 (0.45 to 1.49) |
| Bilateral Implantation | 2.13 (0.92 to 4.90) | 1.68 (0.75 to 3.74) | 1.05 (0.46 to 2.41) |
| *Device Type* |  |  |  |
| Advanced Bionics | Reference group | Reference group | Reference group |
| Cochlear | 0.92 (0.52 to 1.62) | 0.81 (0.48 to 1.36) | 1.00 (0.58 to 1.73) |
| MEDEL | 0.70(0.24 to 2.01) | 0.58 (0.25 to 1.35) | 0.8 (0.31 to 2.05) |

**S2 Table. CI-Specific Model for Maternal Sensitivity.**

* *p* < .05
